# DRAMP 4.0: an open-access data repository dedicated to the clinical translation of antimicrobial peptides

**DOI:** 10.1093/nar/gkae1046

**Published:** 2024-11-11

**Authors:** Tianyue Ma, Yanchao Liu, Bingxin Yu, Xin Sun, Huiyuan Yao, Chen Hao, Jianhui Li, Maryam Nawaz, Xun Jiang, Xingzhen Lao, Heng Zheng

**Affiliations:** School of Life Science and Technology, China Pharmaceutical University, Nanjing 211100, P.R. China; School of Life Science and Technology, China Pharmaceutical University, Nanjing 211100, P.R. China; School of Life Science and Technology, China Pharmaceutical University, Nanjing 211100, P.R. China; School of Life Science and Technology, China Pharmaceutical University, Nanjing 211100, P.R. China; School of Life Science and Technology, China Pharmaceutical University, Nanjing 211100, P.R. China; School of Life Science and Technology, China Pharmaceutical University, Nanjing 211100, P.R. China; School of Life Science and Technology, China Pharmaceutical University, Nanjing 211100, P.R. China; School of Life Science and Technology, China Pharmaceutical University, Nanjing 211100, P.R. China; Mudi Meng Honors College, China Pharmaceutical University, Nanjing 211100, P.R. China; School of Life Science and Technology, China Pharmaceutical University, Nanjing 211100, P.R. China; School of Life Science and Technology, China Pharmaceutical University, Nanjing 211100, P.R. China

## Abstract

Antimicrobial peptides (AMPs) are potential candidates for treating multidrug-resistant bacterial infections, yet only a small number of them have progressed into clinical trials. The main challenges include the poor stability and hemolytic/cytotoxic properties of AMPs. Considering this, in the update of the Data Repository of Antimicrobial Peptides (DRAMP), a new annotation on serum and protease stability is added, and special efforts were made to update the hemolytic/cytotoxic information of AMPs. The DRAMP 4.0 currently holds 30 260 entries (8 001 newly added), consisting of 11 612 general entries, 17 886 patent entries, 96 clinical entries, 377 specific entries, 110 entries with stability data, and 179 expanded entries. A total of 2891 entries possess experimentally determined hemolytic activity information, while 2674 entries contain cytotoxicity data by experimental validation. The update also covers new annotations, statistics, categories, functions, and download links. DRAMP is available online at http://dramp.cpu-bioinfor.org/.

## Graphical Abstract

**Graphical Abstract:**
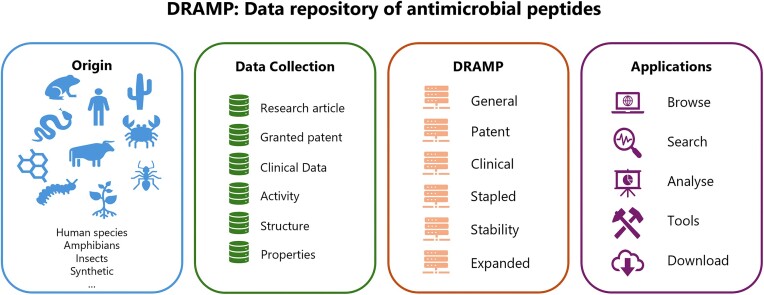

## Introduction

Multidrug-resistant bacteria, widely known as superbugs, have gravely threatened public health (1). Compounding the issue is the scarcity of structural diversity in antimicrobial research and development pipelines (2). Antimicrobial peptides are considered potential candidates for treating multidrug-resistant bacterial infections, due to their broad-spectrum antibacterial activity and multiple mechanisms of action, as well as their relatively difficult to induce drug resistance (3,4). Recent years have seen the breakthrough of high-throughput sequencing and metagenomics, particularly microbiomes, providing substantial resources for AMPs (5,6). The advancement of artificial intelligence (AI) and peptide synthesis technology has also significantly stimulated the design and discovery of AMPs (7,8). To effectively utilize the rapid expansion of AMP information, it is crucial to gather and store data in regularly updating databases. The antimicrobial peptides database APD published in 2008, primarily collects AMPs from natural sources and has been updated to APD3 (9,10). DBAASP, currently in version 3.0, contains 21 957 monomers, 382 multimers, and 236 multi peptides (11). CAMP_R4_ contains 24 243 AMP sequences, 933 structures and 2143 patent AMPs (12). dbAMP2.0 contains 26 447 AMPs and 2262 antimicrobial proteins (13). We constructed the manually annotated database DRAMP, which was published in 2016 and initially contained 17 349 AMPs (14). After regular updates, the last version of DRAMP3.0 contained 22 259 entries (15,16).

Despite the growing concern and the discovery of thousands of AMPs, few of them have progressed to the clinical trials stage (17). In our clinical subset, presently only 96 AMPs were reported to have been engaged in clinical trials. The main obstacles to their clinical translation include significant host toxicity, poor stability *in vivo*, diminished activity in the presence of serum and high production costs (18). Natural AMPs are easily hydrolyzed or degraded by enzymes, leading to a short half-life and rapid clearance *in vivo*. These intrinsic disadvantages of AMPs present both challenges for peptide drug development and opportunities for their design and optimization (19–21).

At present, most AMP databases, such as DBAASP, CAMP, LAMP (22), APD, dbAMP, and our previous version DRAMP3.0 have incorporated hemolytic/cytotoxic annotation, but none of them systematically collected half-life data for AMPs. The database PEPLife (23) is dedicated to collecting peptide half-life information, but it hasn’t been updated since 2016 and is currently inaccessible. In the study, a new annotation of serum stability or half-life has been added in DRAMP4.0.

Compared to the previous version, DRAMP 4.0 includes over 8000 additional entries. Specifically, 110 antimicrobial peptides (AMPs) with experimentally determined stability information have been added. Systematic updates have also been made in sequence modification, classifications and the annotation of cytotoxicity/hemolysis. Additionally, users are encouraged to share their expertise on AMPs, drawing upon published references and credible clinical trials on the submission page.

In adherence to the public sharing protocol required by Scientific Data Journal, we have uploaded the original code on GitHub and peptide data on Figshare, with CC by license for free download and sharing. All the data can be downloaded from the download page, including complete datasheets or sheets categorized by different subsets.

## Materials and methods

### Basic data collection

We used ‘antimicrobial peptides’, ‘host defense peptides’, ‘antiviral peptides’, ‘antibacterial peptides’, ‘antifungal peptides’, ‘anticancer peptides’, ‘stapled antimicrobial peptides’, ‘screened antimicrobial peptides’ and ‘antimicrobial peptides serum stability’ as keywords, searched literature and patents from PubMed (24), Web of Science, China National Knowledge Infrastructure (CNKI), Lens, Clinical Trials.gov (25), and chinadrugtrials.org.cn. These literature and patents are all manually reviewed to extract AMP information and deposited in DRAMP if it fulfills the criteria: (i) each sequence is <100 amino acids in length; (ii) they have unambiguous mature sequences in which precursor and signal regions have been moved. The experimentally determined 3D structures were searched from the Protein Data Bank (PDB) (26), and the PDB IDs were deposited in DRAMP.

AMPs in DRAMP are categorized into four sub-datasets based on their different sources of references: including general dataset, patent dataset, clinical dataset, and specific dataset. The specific dataset contains AMPs with special properties. Currently, it contains four subgroups: (i) stapled AMPs: It is derived from a general dataset, gathering artificial cyclic antimicrobial peptides known as stapled peptides. (ii) Stability dataset: a new subgroup added in DRAMP4.0, dedicated to collecting the stable information, including serum stability, protease stability and half-life *in vitro* or *in vivo*. (iii) Expanded AMPs dataset: a new subgroup added in DRAMP4.0, collected experimental verified AMPs from published Chinese literature. Most of the papers were searched from CNKI and were written in Chinese. Since they lacked PubMed IDs, these AMPs were collected in a separate dataset. (iv) HTS dataset: it includes the AMPs from the high throughput screening (HTS) method, for example, screened by Surface Localized Antimicrobial Display (SLAY) (27).

### Database construction and maintenance

The DRAMP web architecture was based on the platform Linux-Apache-MySQL-PHP. After collecting and screening, all the data were stored in the MySQL database as the back end. The visualization software Navicat for MySQL was used to manage the data. Additionally, HTML, PHP, and JavaScript were applied to develop the front-end web interfaces. The source code of the DRAMP website has been stored in the GitHub public repository. The database is planned to be updated every 2 or 3 months.

**Figure 1.**
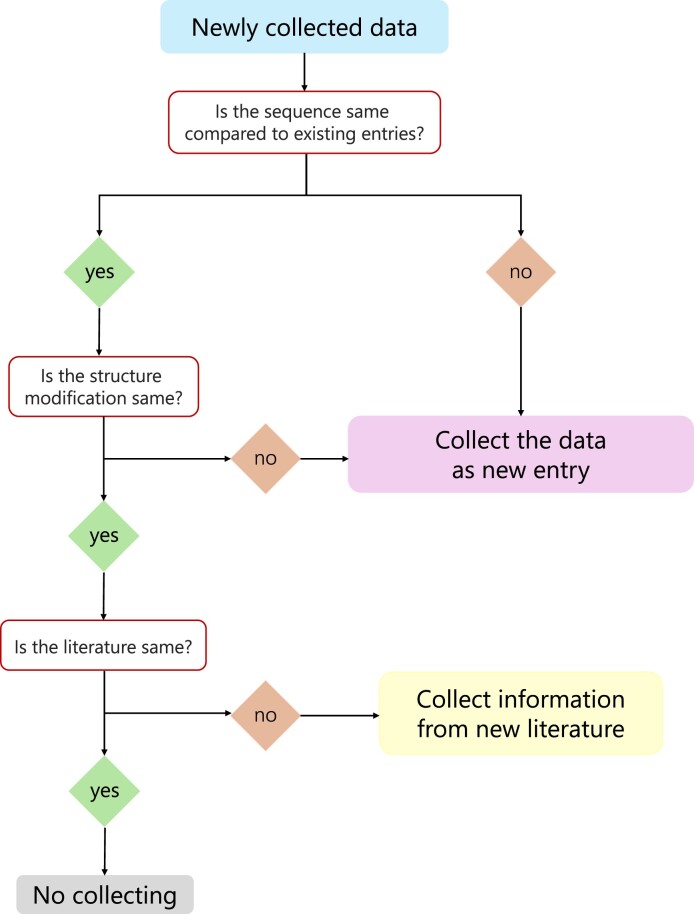
Data update flow chart.

For the daily maintenance and updating of the database, the basic process is as follows: whenever new data are collected, we will first perform sequence comparison, if there are duplicate sequences, we will first determine whether there are different sequence modifications, if there are, they will be included in the database as a new entry, if they are the same, then we will compare the references, if the references are different, we will add the activity, cytotoxicity, hemolytic activity and stability data of the new references to the existing sequence entries. If the sequence is different or the modification is different, the peptide will be included in the database as a new entry. Each updated entry is double-checked by a different person to ensure its accuracy. The flow chart is shown in Figure 1.

### Web architecture

*Home page*. We will regularly review the content of each section on the homepage and make modifications based on the updates. For example, in this update, the NEWS section described the addition of stability information, and the New Released Structures section has been replaced with the latest structural information. Each update will be noted in the NEWS & EVENTS section accordingly.

*Search page*. We provide quick search on the homepage as well as simple search and advanced search on the search page. Quick search can be searched by DRAMP ID, name sequence, and activity as keywords, simple search can be searched by the DRAMP ID, name sequence, and activity as keywords, simple search compared to the quick search, simple search adds simple keywords such as PDB ID, Patent No. and literature title, while advance search enables more accurate searching of AMPs based on multiple keywords (source, 3D structure, toxicity, modifications and so on).

*Browse page*. The browse section was used as a map directed to each sub-dataset, and Stability Data was added to quickly browse the AMPs with experimentally determined stable data. Meanwhile, for each newly collected entry, we grouped them into different sub-categories according to the targeted activity, such as antibacterial, anti-viral, anti-cancer, etc., and put them in the browse list.

*Download page*. All data have been uploaded and are freely available to users.

## Results

### New entries

**Figure 2.**
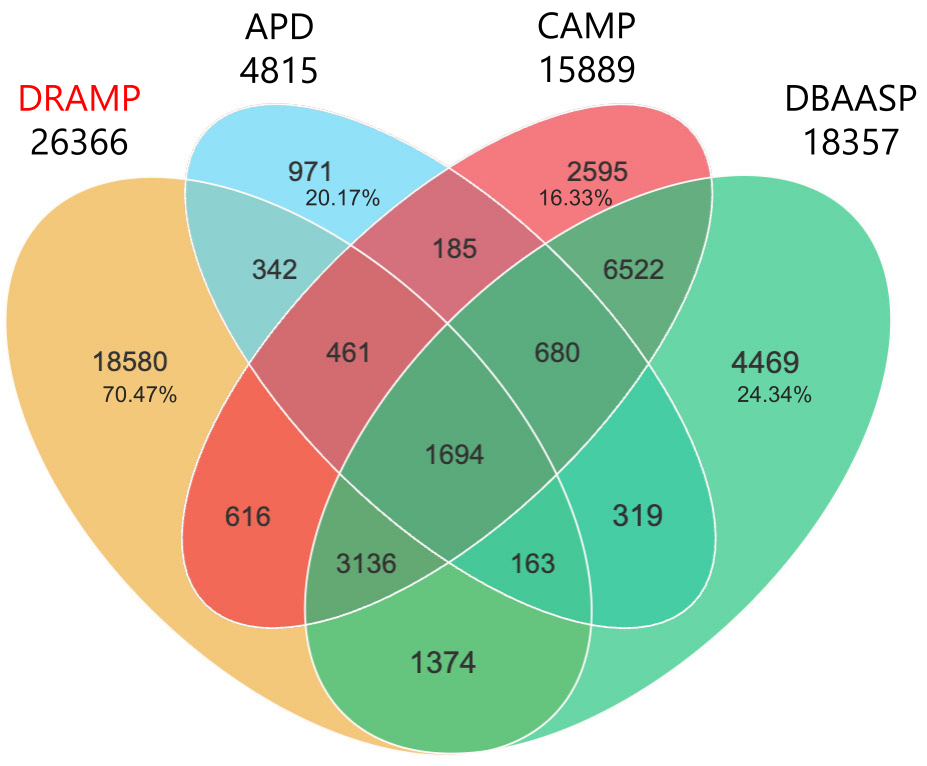
Venn diagram representing the numbers of overlapping and non-overlapping sequences from APD (https://wangapd3.com/main.php), DBAASP (https://dbaasp.org/home), and DRAMP (data as of 1st June 2024). The numbers of nonoverlapping sequences in all four databases were calculated as percentages in corresponding areas. In the statistical process, we excluded redundant sequences or predicted AMPs. Moreover, all peptides in the database with the same sequence but with different structural modifications are considered as one sequence.

In DRAMP 4.0, we added 8001 new entries, including 5 721 general entries, 1 776 patent entries, 19 clinical entries and 196 stapled entries, newly added 110 stability data, and 179 expanded entries, compared to the previous version of DRAMP 3.0. We compared the unique sequences identified from APD (10), DBAASP and DRAMP, following specific criteria outlined in Figure 2. It can be observed that 18 580 entries (70.47%) in DRAMP are unique and not included in other databases. Additionally, it is also evident that certain entries in DBAASP and CAMP are not covered by our DRAMP 4.0. This discrepancy arises from the different inclusion criteria adopted by various databases. DRAMP specifically includes antimicrobial peptides that are no longer than 100 amino acids (AA) in length, whereas the other two databases do not have explicit restrictions on the length of the peptides they include.

### New classification

**Figure 3.**
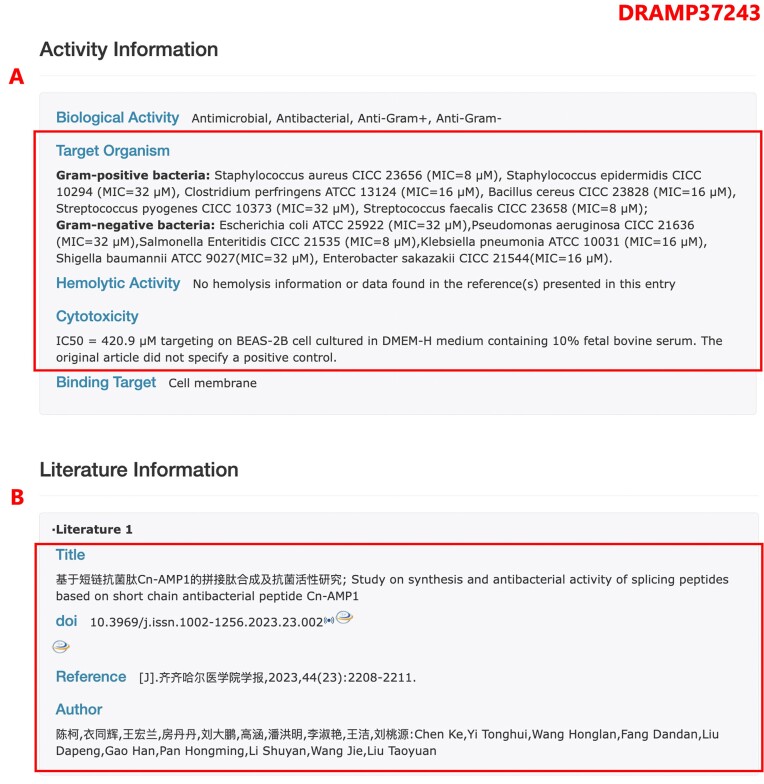
The page of entry DRAMP37243 in the expanded AMPs dataset. (**A**) Screenshot of the activity information section, all the activity data have been translated into English. (**B**) Screenshot of the literature information, since Chinese literature generally does not have PubMed_ID, in this section, we only provide information such as article titles and authors in both Chinese and English.

In DRAMP 4.0, we have added the Expanded AMPs dataset, which comprises experimentally verified AMPs from Chinese literature. Due to the publication of these AMPs in Chinese papers, their information has not been indexed or recorded in contemporary antimicrobial peptide databases such as DBAASP, CAMP, APD and others. Consequently, it has been challenging for researchers outside of China to access and utilize this data. To promote the sharing of these data, we have diligently compiled experimental data on antimicrobial peptides from Chinese literature (Figure 3). This compilation can serve as a valuable resource for the design and application of novel antimicrobial peptides.

### New annotations

*Stability information*. Despite the growing concern and the discovery of thousands of AMPs, few of them have progressed to the clinical trials stage. Although antimicrobial peptides (AMPs) were discovered in the last century, only a few peptide antibiotics, such as vancomycin, daptomycin, polymyxin, and teicoplanin, have been approved for clinical use. The clinical application of AMPs remains significantly limited due to issues such as toxicity, stability, and production costs. To further promote the clinical translation of AMPs and assist researchers in designing and selecting effective AMP drugs, DRAMP 4.0 includes the collection of AMP entries with experimentally validated stability data not yet included in any existing online AMP databases. The stability data section provides three types of annotations: the type of serum or protease, stability data, and methods used for stability determination, along with links to relevant literature.

**Figure 4.**
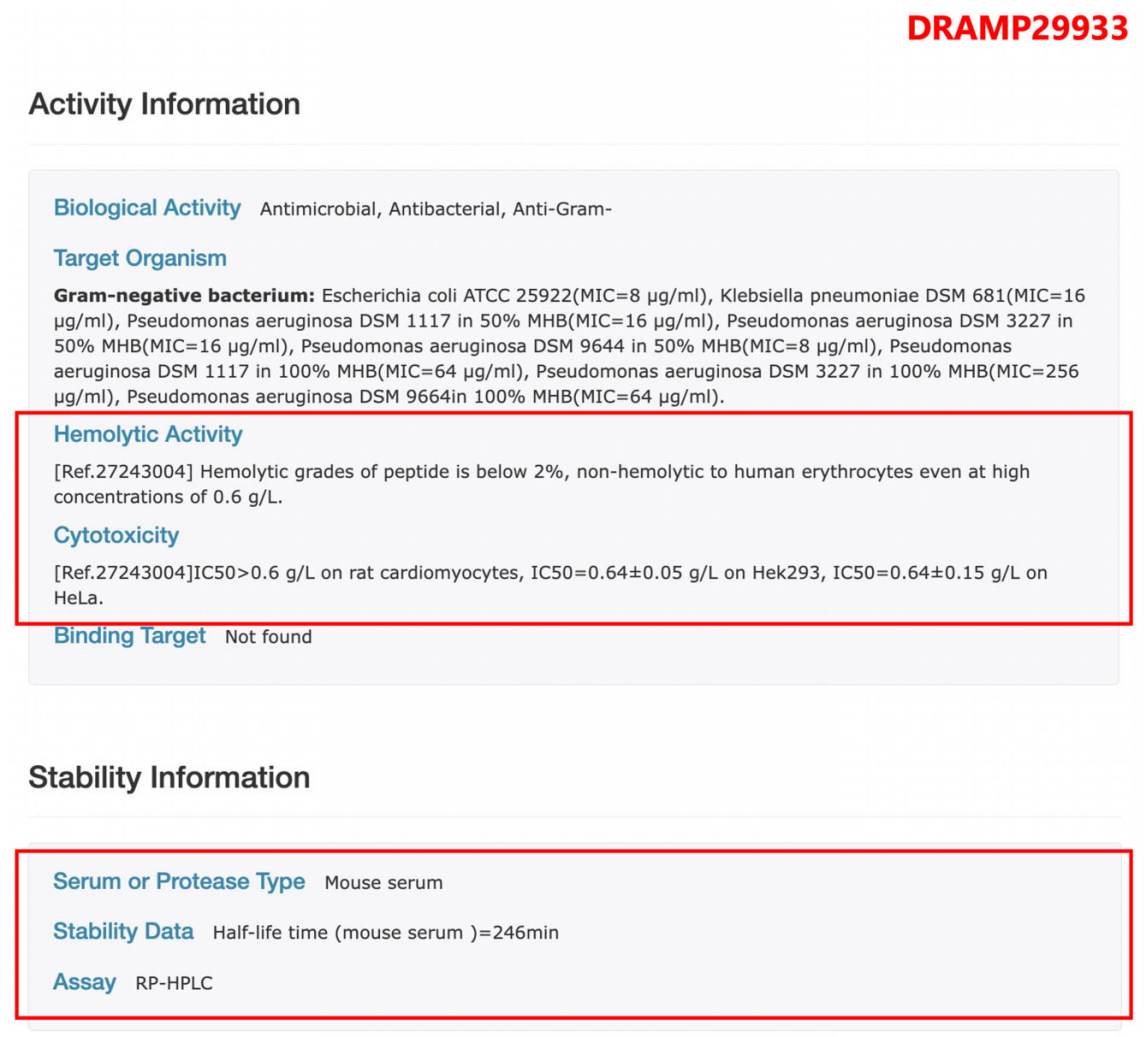
The page of entry DRAMP29933 in the stability dataset, the AMP’s stability is determined by RP-HPLC (Reverse Phase High Performance Liquid Chromatography) in mouse serum, and the half-life is 246 min.

As illustrated in Figure 4, we have newly incorporated experimentally determined serum stability data. For instance, the half-life of the DRAMP29933 peptide in mouse serum is 246 min, as measured by the RP-HPLC method. Additionally, we have compiled hemolytic and/or cytotoxic data of AMPs. In this update, we have added 1421 hemolytic activity data and 2611 cytotoxicity data.

### Reorganization

**Figure 5.**
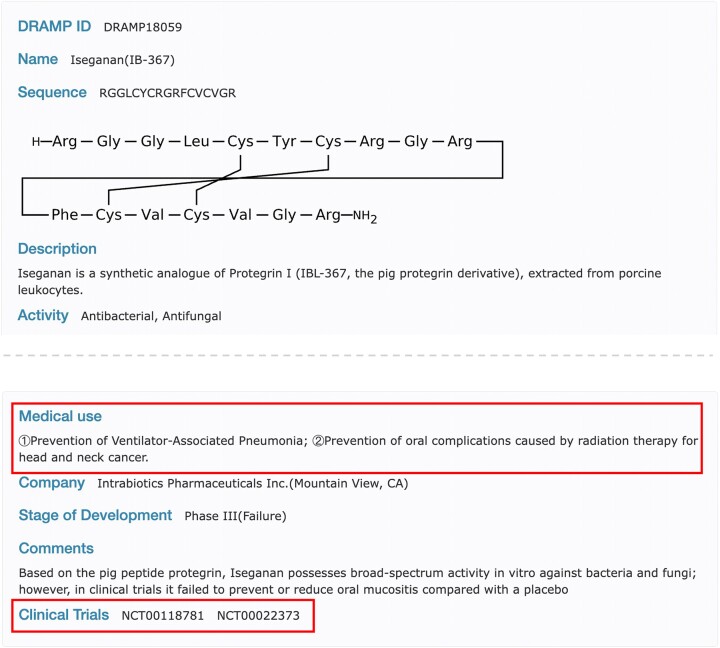
Screenshot of the detailed page of DRAMP18059 in the clinical dataset, where the red box represents newly added or modified content.

*Clinical dataset*. In previous versions, clinical antimicrobial peptides (AMPs) were stored as separate entries based on different clinical indications. This approach led to redundant sequences in the clinical data, as the same AMP might be used to treat multiple infectious diseases. To ensure the uniqueness of AMP entries, the current update has consolidated entries with identical sequences. Different clinical indications are now listed under the ‘Medical Use’ field, clearly delineating the various clinical applications of the peptide for users. Additionally, a new ‘Clinical Trials’ field has been introduced to document the trial identification numbers associated with the drug in clinical trial databases. For example, as Figure 5 shows, Iseganan (DRAMP18059) is used for the prevention of ventilator-associated pneumonia and oral complications arising from radiotherapy in head and neck cancer. Corresponding clinical trials were recorded in the ClinicalTrials database under the identifiers NCT00118781 and NCT00022373.

### Download links

**Figure 6.**
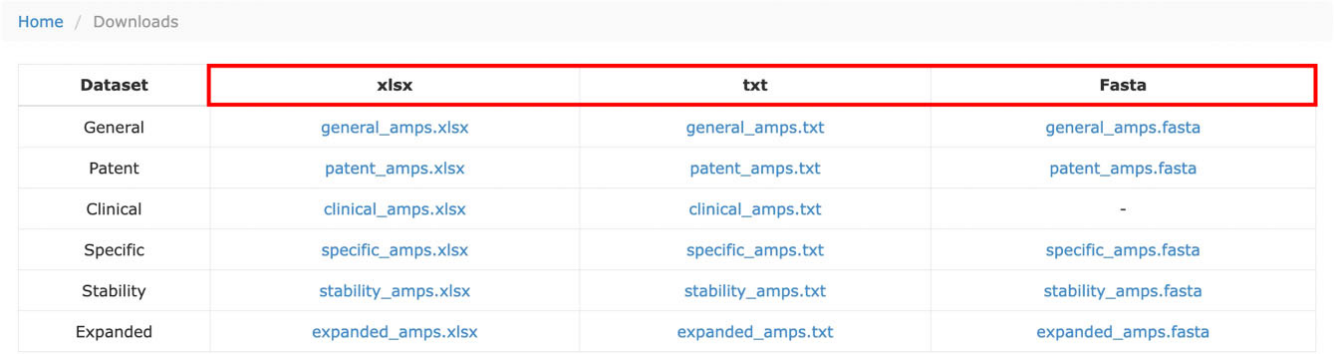
Screenshots of the download page.

To meet the diverse needs of users, three file formats (Fasta, xlsx, txt) are available for download on the download page, as illustrated in Figure 6. Additionally, categorized download links are provided for antimicrobial peptides with different activities, including the newly added category for Anti-SARS-CoV-2 data.

## Discussion

Antimicrobial peptides are considered potential candidates for treating multidrug-resistant bacterial infections, for they have broad antibacterial activity and are relatively difficult to induce drug resistance. However, due to limitations such as cytotoxicity, low stability, and hemolytic activity *in vivo*, only a few AMPs have successfully been developed into drugs.

We constructed DRAMP and published it in 2016 (14). By 2019, the database expanded significantly with the addition of 2 550 new entries, bringing the total AMP collection to 19 899 entries. Consequently, DRAMP 2.0 has become a widely used resource in the field of antimicrobial peptides research (15). The update in 2021 added 2360 new entries, including a specific subgroup of 181 stapled AMPs, making a total number of 22 259 entries in DRAMP3.0 (16). The current update, DRAMP4.0 contains 30 260 entries (8001 newly added), consisting of 11 612 general entries, 17 886 patent entries, 96 clinical entries, 377 stapled entries, 110 stability data and 179 expanded entries. It is worth noting that there has been a significant expansion in the number of antiviral peptides (3645 more than the previous version). This may be due to the growing research on antivirals, driven by the global COVID-19 pandemic.

In DRAMP 4.0, a dedicated new annotation of serum or proteinase stability is added, to gather the scattered data for the design of stable AMPs. We also specifically collected data on antimicrobial peptide hemolysis and toxicity and reorganized the clinical database, to promote the clinical translation of AMPs. Additionally, in the clinical dataset, we have included clinical trial identification numbers of ClinicalTrials.org, making it easy for users to track the progress of clinical research. Moreover, a specialized AMPs expanded subset has been created to collect AMP data documented in Chinese literature, to promote the research and development of antimicrobial peptides.

Recent updates to the DRAMP have been released on the website, including new statistical data and preliminary analyses presented through charts and tables. Crucially, all data and annotations within DRAMP are now available for download via provided links. The specific updates included in this version are detailed in Table 1.

**Table 1.** The update list of DRAMP 4.0 comparing to DRAMP 3.0

Update class	Classification	Sub classification	DRAMP 3.0	DRAMP 4.0
Entry	General dataset	Anti-Gram+	2452	3071
		Anti-Gram-	2618	2918
		Antifungal	1802	3516
		Anticancer	149	5906
		Antiviral	215	3860
		Antiprotozoal	17	17
		Antibiofilm	44	44
		Anti-inflammatory	4	4
	Patent dataset		16 110	17 886
	Clinical dataset		77	96
	Stapled dataset		181	377
	Stability data			110
	Expanded AMPs			179
Structure	PDB structure		283	419
	Predicted structure		263	1570
Annotation field	Hemolytic activity		1 470	2891
	cytotoxicity		63	2674

DRAMP is committed to ongoing data collection, regular updates to existing entries, and the incorporation of additional functionalities to support the rational design and research of AMPs.

## Data Availability

Users can access DRAMP 4.0 (http://dramp.cpu-bioinfor.org/downloads/) for the data. DRAMP is licensed under a Creative Commons Attribution 4.0 International License (CC BY 4.0) and allows download and use of the data freely. An Excel document recording sequences from four databases, two Python scripts for statistics and drawing, and the source codes of DRAMP are available in GitHub, Zenodo (DOI is 10.5281/zenodo.13938020). The peptide data is also available on Figshare (DOI 10.6084/m9.figshare.27233508).
